# Biliary Tract Abnormalities as a Cause of Distal Bowel Gas in Neonatal Duodenal Atresia

**DOI:** 10.1155/2018/8041427

**Published:** 2018-06-26

**Authors:** Surasak Puvabanditsin, Marissa Botwinick, Charlotte Wang Chen, Aditya Joshi, Rajeev Mehta

**Affiliations:** Department of Pediatrics, Rutgers Robert Wood Johnson Medical School, One Robert Wood Johnson Place, New Brunswick, NJ 08903, USA

## Abstract

**Background:**

The presence of distal bowel gas in an infant does not exclude the diagnosis of duodenal atresia.

**Case Presentation:**

We report a term neonate with Down syndrome. The infant developed vomiting and cyanosis with each feeding soon after birth. Plain film abdominal X-rays showed a nonspecific gas-filled stomach and small bowel. Duodenal atresia and an anomalous common bile were noted on an upper GI study and exploratory laparotomy.

**Conclusion:**

In the absence of a “double bubble” appearance and intestinal gas distally on a plain radiograph, one must not exclude duodenal atresia as the differential diagnosis.

## 1. Introduction

In the newborn with duodenal atresia, the hallmark of an abdominal radiograph is the “double bubble” with a gaseous distension of the stomach and proximal duodenum and the total absence of gas in the distal bowel.

Only 23 cases of duodenal atresia with an anomalous common bile duct have been reported in the literature. We present a case of a Down syndrome neonate with duodenal atresia and gas in the distal intestine without a “double bubble” sign.

## 2. Case Report

**Figure 1:**
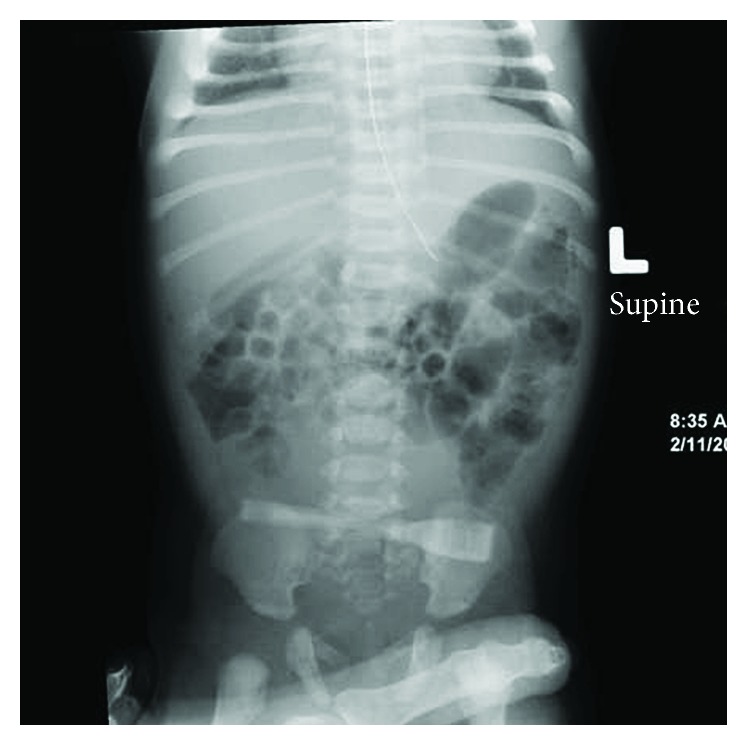
Abdominal radiograph showing an air-filled stomach, duodenum, and jejunum.

**Figure 2:**
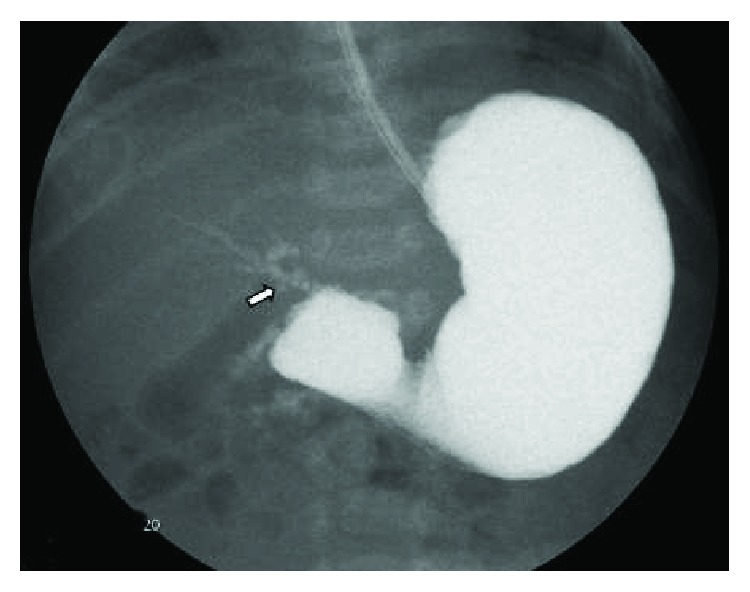
Upper gastrointestinal series showing complete obstruction to the flow of contrast at the second portion of the duodenum. There is also contrast filling of the biliary tree above the duodenal bulb noted (arrow).

**Figure 3:**
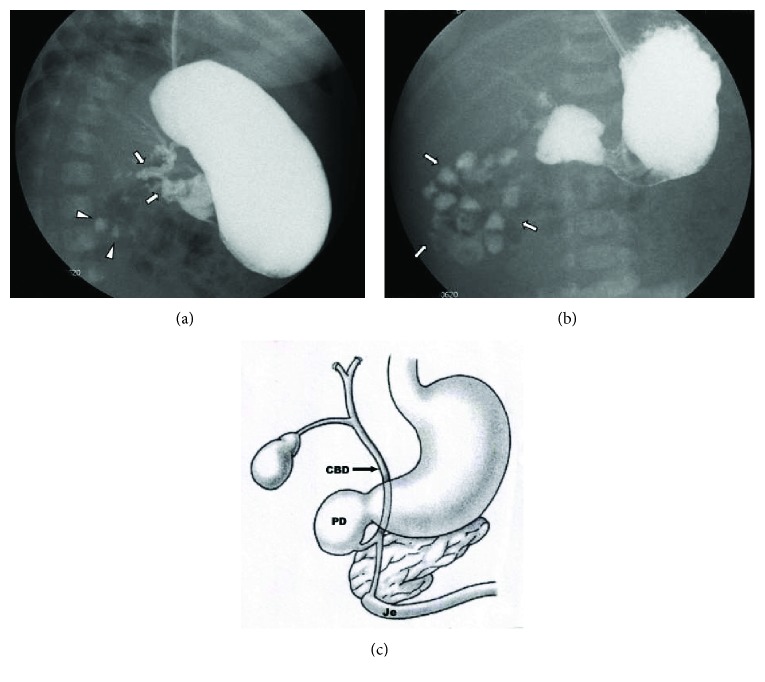
(a) Upper gastrointestinal series showing a complete obstruction of the duodenum and contrast filling of anomalous bifurcated bile ducts (arrows). The small contrast was also noted in the distal bowel (arrowheads). (b) Upper gastrointestinal series showing a complete obstruction at the second portion of the duodenum, and contrast was seen in the proximal jejunum which is located in the right upper quadrant. The proximal location of the jejunum indicates a malrotation of the intestine without evidence of a small bowel obstruction. (c) Diagram showing biliary tract abnormality associated with duodenal atresia (PD—proximal duodenum, Je—jejunum, and CBD—common bile duct).

A term female was born at 39 weeks of gestation to a 32-year-old G2P1 by spontaneous vaginal delivery. Apgar scores were 9 and 9 at 1 and 5 minutes, respectively. The pregnancy was uncomplicated. Physical examination revealed a weight of 3650 gm (70th centile), length of 51 cm (60th centile), and head circumference of 33 cm (15th centile). The infant had features of Down syndrome: flattened facies, upslanting palpebral fissures, palmar creases, and sandal gap deformities of the great and second toes. Karyotype was obtained on the first day of life. Recurrent vomiting after each feeding was noted since birth. A plain abdominal radiograph showed a nonspecific bowel gas pattern with gas noted in the stomach, duodenum, and distal bowel (Figure 1). An upper gastrointestinal (UGI) series showed a complete obstruction to the flow of barium at the proximal portion of the duodenum. A small amount of contrast was also seen to exit from the proximal duodenal segment into a biliary duct structure with a retrograde filling of the biliary tree into the intrahepatic system as well as into the gallbladder through the cystic duct. The contrast was also seen in the proximal jejunum which was located in the right upper quadrant (Figures 2 and 3). The patient underwent exploratory laparotomy on the 3rd day of life. Duodenal atresia was repaired. Malrotation was identified, and a Ladd procedure and appendectomy were performed. The postoperative course was uneventful, and the infant was discharged home at 35 days of life. Karyotype confirmed the diagnosis of trisomy 21 (Down syndrome).

## 3. Discussion

Duodenal atresia (DA) occurs in approximately 1 in 2500–7500 live births without a sex-associated difference. Approximately 25–40% of infants with duodenal atresia have trisomy 21 (Down syndrome). Approximately 8% of infants with Down syndrome have duodenal atresia [1–3]. There is also an association of VACTERL anomalies (vertebral, anorectal, cardiac, tracheoesophageal, renal, and limb anomalies). The classic abdominal X-ray depicts the “double bubble” which represents the air-filled stomach and obstructed duodenum and the absence of distal bowel gas [3]. During pregnancy, duodenal atresia can cause an increase of fluid in the amniotic sac resulting in polyhydramnios; this may be the first sign of a DA. A double bubble can be seen with prenatal ultrasound, in which case the bubbles are filled with fluid. This appearance should be interpreted with caution as transient double bubbles can result from transient duodenal fluid accumulation and slow peristalsis, and these have been observed in fetuses subsequently found to be healthy [3–5]. After delivery, an infant with duodenal atresia generally has a scaphoid abdomen but one may occasionally observe epigastric fullness from dilation of the stomach and proximal duodenum. The passage of meconium within the first 24 hours of life is not usually altered.

Unlike more distal small bowel (jejunal/ileal) atresia which is believed to be caused by an ischemic episode, DA is believed to result from the failure of the recanalization of the bowel lumen following the phase of epithelialization, proliferation, and subsequent vacuolization of the alimentary tract during embryonic development [6–8]. Between the 30th and 60th days of embryonic development, growth and differentiation of the alimentary tract are proceeding at great speed. In a matter of a week or 10 days, its cross-sectional area increases eighty-fold. At the beginning of the sixth week, the duodenum contains a small lumen. From the 7th to 8th weeks, the patency of the duodenal lumen is restored by a coalescence of vacuoles, which produces 2 parallel channels in the duodenum, and at the same time, 2 channels appear in the developing biliary system [7–9]. This is the time when genetic and environmental influences would exert their maximal effect. It is not unreasonable to suppose that during this period, duodenal atresia may develop.

An anomalous bile duct termination may occur when atresia develops between the 2 orifices of the bile ducts. Boyden et al. [10], in discussing the abnormalities which may occur at the entrance of the common bile duct and pancreatic ducts, stated that they were the result of an embryological “traffic jam” [8, 10]. During development, two blindly ending channels appear in the duodenum at the stage of vacuolization and at the same time two channels also appear in the developing biliary system. Each of these joins up separately with the developing duodenal channels. If some accident affected the “embryological events” at this stage of development, aberrations of the lower biliary tract could be expected in association with duodenal atresia. The atretic segment is an abnormally sited bile channel [8, 11]. In the report by Komuro et al. [12], 12 (22.2%) of 54 cases with duodenal atresia and 9 cases with and 3 without distal bowel gas had an anomalous bifurcated bile duct conduit between the proximal and distal segments.

In summary, duodenal atresia with a plain radiograph showing distal bowel gas via anomalies of the bile duct is more common than initially thought. We describe a case with findings that are contradictory to the cardinal sign of duodenal atresia namely the “double bubble” with the absence of gas in the distal intestine. Prenatal sonographic findings suggestive of duodenal atresia should not be dismissed based on plain film findings.
